# Technical and Target Lesion Failure in Calcified Coronary Lesions: Residual Risk Beyond Successful Lithotripsy

**DOI:** 10.1016/j.jacadv.2026.103010

**Published:** 2026-07-15

**Authors:** Federico Oliveri, Martijn.J.H. Van Oort, Akshay Phagu, Brian O. Bingen, Valeria Paradies, Gianluca Mincione, Bimmer E. Claessen, Ioannis Karalis, Maria Mouratidou, Aukelien C. Dimitriu-Leen, Marios Sagris, Joelle Kefer, Tessel Vossenberg, Alessandro Mandurino-Mirizzi, Frank Van der Kley, J. Wouter Jukema, Ibtihal Al Amri, José Montero-Cabezas

**Affiliations:** a Department of Cardiology, Leiden University Medical Center, Leiden, the Netherlands; b Department of Cardiology, Maasstad Ziekenhuis, Rotterdam, the Netherland; c Department of Cardiovascular Medicine, Humanitas Research Hospital-IRCCS, Rozzano, Italy; d Department of Cardiology, Amsterdam University Medical Center, the Netherlands; e Department of Cardiology, Red Cross Hospital, Athens, Greece; f Department of Cardiology, Radboud University Medical Center, the Netherlands; g Department of Cardiology, Hippokration Hospital, Athens, Greece; h Department of Cardiology, Saint-Luc Bruxelles, Belgium; i Department of Cardiology, Frisius Medisch Centrum, the Netherlands; j Department of Cardiology, University of Salento, Lecce, Italy; k The Netherlands Heart Institute, Utrecht, the Netherlands

**Keywords:** calcified coronary lesions, coronary calcification, intracoronary lithotripsy, IVL and target lesion failure, target lesion failure

## Abstract

**Background:**

Intravascular lithotripsy (IVL) has been demonstrated effective and safe in the treatment of balloon-crossable calcified coronary lesions. However, a significant subset of patients still experiences suboptimal technical results and subsequent target lesion failure (TLF), leaving a gap in our understanding of procedural success drivers.

**Objectives:**

This study sought to identify factors independently associated with technical failure and 12-month TLF in calcified coronary lesions treated with IVL-assisted percutaneous coronary intervention.

**Methods:**

From the multicenter BENELUX-IVL registry (NCT06577038), we included patients with complete quantitative coronary analysis. The primary technical endpoint was technical failure (unsuccessful IVL delivery, residual stenosis >30%, or procedural complications). The primary clinical endpoint was 12-month TLF (cardiac death, target-vessel myocardial infarction, or ischemia-driven revascularization).

**Results:**

A total of 571 patients (including 595 target lesions) were included. Technical failure occurred in 60 (10.5%) of the lesions, whereas 12-month TLF was reported in 41 (7.2%) cases. Multivariable analysis identified multivessel disease (OR: 1.39; 95% CI: 1.02-1.90), in-stent lesions (OR: 1.94; 95% CI: 1.14-3.30), and the absence of intravascular imaging (OR: 1.95; 95% CI: 1.13-3.40) as independent determinants of technical failure. Left circumflex (HR: 2.97; 95% CI: 1.49-5.92), in-stent target lesions (HR: 2.01; 95% CI: 1.02-3.52), technical failure (HR: 2.42; 95% CI: 1.26-4.61), diabetes mellitus (HR: 2.35; 95% CI: 1.23-4.47), reduced estimated glomerular filtration rate (HR: 0.98; 95% CI: 0.97-0.99), and younger age (HR: 0.95; 95% CI: 0.92-0.98) emerged as factors independently associated with TLF.

**Conclusions:**

Our findings demonstrate that residual risk in IVL-assisted percutaneous coronary intervention is driven by a distinct interplay of modifiable and nonmodifiable factors, rather than inherent limitations of the technology itself.

Moderate to severe coronary artery calcification (CAC) is present in up to 25% to 30% of patients undergoing percutaneous coronary intervention (PCI).^1^^,^^2^ CAC frequently hinders successful device delivery and adequate vessel dilatation, increasing the odds of stent underexpansion and malposition, ultimately resulting in higher rates of stent thrombosis and target lesion failure (TLF).^3^ The rapid evolution of calcium-modifying technologies has provided several dedicated devices that have dramatically increased procedural success, reduced complications, and improved long-term clinical outcomes.^4^ Among these, intravascular lithotripsy (IVL) has emerged as a highly effective and safe option for treating balloon-crossable calcified lesions.^5^ By delivering sonic pressure waves, IVL induces transmural calcium fractures, thereby increasing vessel compliance and facilitating optimal stent expansion (SE) with a remarkably low rate of procedural complications.^6^^,^^7^ Despite the consistently high procedural success rates demonstrated in both pivotal studies and real-world registries, there remains a subset of patients in whom IVL-assisted PCI still results in suboptimal procedural results and consequently higher risk of TLF.^5^^,^^8^ Identifying the determinants of technical failure and TLF is crucial to risk-stratify patients upfront, optimize patient selection, and tailor adjunctive treatment strategies. Currently, these specific determinants are not fully understood. Therefore, the aim of our study was to identify the determinants of technical and TLF in calcified coronary lesions treated with IVL.

## Methods

### Population and data collection

The BENELUX-IVL registry (NCT06577038) is an international, multicenter, prospective study enrolling all-comer patients aged ≥18 years who underwent IVL during PCI. To objectively evaluate procedural results, we selected participants from the overall cohort with complete pre-PCI and post-PCI quantitative coronary analysis (QCA). Cases were excluded if they presented suboptimal angiographic image quality (eg, severe foreshortening, excessive vessel overlap, or poor contrast opacification) or if two near-orthogonal projections were not available for accurate assessment. All procedures utilized the Shockwave Intravascular Lithotripsy Coronary System (Shockwave Medical). Technical decisions, including timing, IVL balloon sizing, pulse delivery, and maximum pressure, as well as the use of adjunctive debulking devices or intracoronary imaging (ICI), were left to the operator's discretion and systematically recorded. Demographic, procedural, and follow-up data were extracted from institutional electronic health records. Patients unable to provide informed consent were excluded. Angiographic and imaging data were analyzed at a centralized core laboratory, and the study protocol was approved by the local ethics committees of all participating institutions.

### Definitions and imaging analysis

In accordance with the latest European Association of Percutaneous Cardiovascular Interventions consensus, coronary calcification severity was assessed using established intravascular imaging criteria. For intravascular ultrasound (IVUS), severe calcification was defined by a 360° calcium arc, an arc >270° extending ≥5 mm in length, the presence of a calcified nodule, or a vessel diameter <3.5 mm. For optical coherence tomography (OCT), a validated scoring system was applied: 2 points for a maximum calcium angle >180°, 1 point for a maximum thickness >0.5 mm, and 1 point for a longitudinal length >5 mm.^9^, ^10^, ^11^ In the absence of intravascular imaging, severe calcification was defined by the presence of radiopaque densities visible without cardiac motion before contrast injection, involving both sides of the arterial wall.^9^ Alternatively, the "dog-bone effect”, characterized by the underexpansion of the mid-portion of a 1:1 non-compliant balloon at high inflation pressures, was considered an indication for IVL. Vessel and stent characteristics were retrospectively evaluated offline using QCA and intravascular imaging. QCA was performed pre-IVL and post-IVL using the Medis Suite QCA (2D/3D) software (version 4.0.24.4; Medis Medical Imaging System BV). Analysis of IVUS and OCT were performed using QCU-CMS 4.69 (Leiden University Medical Center). ICI parameters were evaluated based on the European Association of Percutaneous Cardiovascular Interventions consensus on the clinical use of ICI.^12^ In detail, ICI measurements included the reference vessel diameter, area stenosis, pre-PCI minimum lumen area, max Ca^2+^ angle, and post-PCI minimum stent area (MSA). The asymmetricity index was calculated by subtracting the minimum lesion diameter from the maximum lesion diameter at the minimum lumen area or MSA, and then dividing this difference by the maximum lesion diameter. SE at the MSA was assessed by dividing the MSA by the reference vessel area.

### Study endpoints

The primary technical endpoint was technical failure, defined as the occurrence of at least 1 of the following: 1) unsuccessful delivery of the IVL catheter across the target lesion; 2) residual diameter stenosis >30%, as assessed by QCA; and 3) procedural coronary complications (eg, flow-limiting dissection, perforation, and final TIMI flow grade <3). The primary clinical endpoint was TLF at 12 months, defined as a composite of cardiac death, target-vessel myocardial infarction (MI), or ischemia-driven target lesion revascularization.

### Statistical analysis

Continuous variables are reported as mean ± SD or median with IQR (25th–75th percentile), depending on their distribution. Normality was assessed through the visual inspection of Q-Q plots. Categorical variables are expressed as absolute frequencies and percentages. A preliminary bivariate correlation matrix (using Pearson’s or Spearman’s coefficients, as appropriate) was generated to explore associations between clinical, procedural, and angiographic variables. To identify determinants of technical failure and 12-month TLF, univariate binary logistic regression and cox regression were respectively performed. Multivariable models were constructed by including variables considered clinically relevant or biologically plausible a priori, as well as variables showing significant univariable associations when these associations were clinically interpretable and biologically plausible. Variable selection was therefore not based solely on univariable statistical significance, and automated stepwise or backward selection procedures were not used. The number of variables included in the multivariable models was restricted according to the number of observed outcome events to reduce the risk of overfitting. Multicollinearity was assessed using the variance inflation factor, with a threshold of <10 indicating no significant redundancy. Logistic regression results are reported as ORs, and Cox regression results as HRs, both with 95% CIs. The cumulative incidence of TLF-free survival over the 12-month follow-up period was estimated using the Kaplan-Meier method, and a number-at-risk table was provided to illustrate the temporal distribution of clinical events. Forest plots were created to visualize the associations between the evaluated variables and TLF. A *P* value <0.05 was considered statistically significant. Statistical analyses were performed using SPSS (version 25.0; IBM) and R (version 4.4.3; R Core Team, 2024).

## Results

### Baseline characteristics

A total of 571 patients with calcified coronary lesions treated with IVL and with complete preprocedural and postprocedural QCA were included in the analysis (Table 1). The median age was 74 years (IQR: 68-80), and 26.4% were women. The median SYNTAX score was 20 (IQR: 12-29). The most common clinical presentation was stable angina (50.8%), followed by non–ST-segment elevation MI (25.3%), unstable angina (9.1%), ST-segment elevation MI (6.7%), and other indications (n = 46, 8.1%).

### Procedural characteristics

A total of 595 lesions were treated (Table 2). Procedures were predominantly performed via radial access (75.6%), with a median procedural duration of 81 minutes (IQR: 60-111) and a median contrast volume of 160 mL (IQR: 130-180). The left anterior descending artery was the most frequently treated vessel (41.1%), followed by the right coronary artery (33.8%), circumflex artery (13.9%), and left main (10.3%). In-stent lesions accounted for 27.6% of cases, bifurcations for 21.7%, ostial lesions for 24.0%, and chronic total occlusions for 8.6%. Rotational atherectomy before IVL was used in 12.3% of cases. Stenting was performed in 92.3% of cases, whereas 7.7% were treated with drug-coated balloons alone. ICI was used in 311 patients (54.5%), predominantly with IVUS (92.9%). Intraprocedural complications occurred in 35 patients (6.1%), including severe dissections in 10 (1.8%), abrupt vessel closure in 9 (1.6%), perforation in 8 (1.4%), and hemodynamic instability in 8 (1.4%). IVL-related complications were infrequent (0.9%).

### Procedural and clinical outcomes

Technical success was achieved in 511 patients (89.5%), corresponding to a technical failure rate of 10.5%. At 12 months, TLF occurred in 41 patients (7.2%). Cardiac death occurred in 17 patients (4.6%), target-vessel MI in 17 (4.6%), and ischemia-driven target lesion revascularization in 36 (6.3%) (Table 3).

### Regression analysis

**Figure 1:**
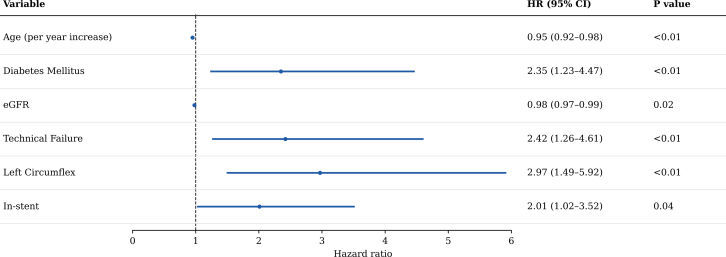
Forest Plot of Factors Associated With 12-Month Target Lesion Failure eGFR = estimated glomerular filtration rate.

On univariate logistic regression analysis, a higher number of diseased vessels, the presence of in-stent lesions, and the absence of ICI were associated with technical failure, whereas long lesions (>20 mm) were inversely associated. In the multivariable analysis, multivessel disease (OR: 1.39; 95% CI: 1.02-1.90; *P* = 0.035), in-stent lesions (OR: 1.94; 95% CI: 1.14-3.30; *P* = 0.015), and absence of ICI (OR: 1.95; 95% CI: 1.13-3.40; *P* = 0.017) remained independent determinants of technical failure (Table 4). Left circumflex (HR: 2.97; 95% CI: 1.49-5.92), in-stent target lesions (HR: 2.01; 95% CI: 1.02-3.52), technical failure (HR: 2.42; 95% CI: 1.26-4.61), diabetes mellitus (DM) (HR: 2.35; 95% CI: 1.23-4.47), reduced estimated glomerular filtration rate (HR: 0.98; 95% CI: 0.97-0.99), and younger age (HR: 0.95; 95% CI: 0.921-0.989) emerged as factors independent factors independently associated with TLF predictors (Figure 1, Table 5).

**Table 1:** Baseline Characteristics (N = 571)

Age, y	74 [68-80]
Female	151 (26.4)
BMI	26.4 [23.9-29.0]
Hypertension	404 (70.8)
Dyslipidemia	311 (54.4)
Smoking history	283 (49.6)
Diabetes mellitus	191 (33.4)
Premature FH of CAD^a^	139 (24.3)
PAD	80 (14.0)
LVEF	53 [41-55]
Syntax score	20 [12-29]
Chronic kidney disease (eGFR <60 mL/min/1.73 m^2^)	171 (29.9)
GFR (mL/min)	71 [53-85]
Previous PCI	256 (44.8)
Previous CABG	99 (17.3)
Previous MI	205 (35.9)
Previous stroke/TIA	69 (12.1)
Clinical presentation	
Stable angina	290 (50.8)
Unstable angina	52 (9.1)
NSTEMI	145 (25.3)
STEMI	38 (6.7)
Others	46 (8.1)
Angina pectoris^b^	
Class I	31 (5.4)
Class II	245 (42.9)
Class III	149 (26.1)
Class IV	55 (9.6)
Angina equivalent/unknown	91 (15.9)
Antiischemic medication	
Beta blockers	348 (60.9)
Nitrates	155 (27.1)

BMI = body mass index; CABG = coronary artery bypass graft; CAD = coronary artery disease; FH = family history; GFR = glomerular filtration rate (using the MDRD [Modification of Diet in Renal Disease] formula); LVEF = left ventricular ejection fraction; NSTEMI = non ST-elevation myocardial infarction; PAD = peripheral artery disease; PCI = percutaneous coronary intervention; STEMI = ST-segment elevation myocardial infarction; TIA = transient ischemic attack.

Values are mean ± SD, median (IQR), or n (%).

a Family history of CAD before 50 years-old.

b According to the Canadian Cardiovascular Society grading of angina pectoris.

**Table 2:** Procedural Characteristics (N = 571)

Procedural time (min)	81 [60-111]
Contrast volume (mL)	160 [130-180]
Access	
Radial	432 (75.6)
femoral	137 (24.0)
brachial	2 (0.4)
Target lesion	595
Left main	61 (10.3)
Left anterior descending artery	245 (41.1)
Circumflex	83 (13.9)
Right coronary artery	201 (33.8)
Arterial graft	0 (0)
Venous graft	5 (0.9)
Bifurcation	124 (21.7)
CTO	49 (8.6)
In-stent	158 (27.6)
Ostial lesions	137 (24.0)
Long lesions (>20 mm)	399 (69.9)
Need for mechanical support	17 (3.0)
IABP	2 (0.4)
Impella	13 (2.3)
VA-ECMO	3 (0.5)
Rotational atherectomy (before IVL)	70 (12.3)
Cutting balloon (before IVL)	5 (0.9)
Pre-IVL largest balloon (mm)	3.5 [3.0-4.0]
Pre-IVL high-pressure dilatation (atm)	521 (90.2)
Pre-IVL maximum pressure dilatation (atm)	20 [16-22]
IVL balloon crossing success	560 (98.1)
IVL pulses delivered	
<80	263 (66.1)
80	280 (49.0)
>80	28 (4.9)
IVL maximum balloon diameter (mm)	3.5 [3.0-4.0]
Post-IVL high-pressure dilatation (atm)	520 (91.0)
Post-IVL largest balloon (mm)	3.0 [3.0-3.5]
Post-IVL maximum pressure dilatation (atm)	20 [18-22]
Total stent length (mm)	38 [24-59]
Stent maximum diameter (mm)	3.5 [3.5-4.0]
Treatment after IVL	
Stenting	527 (92.3)
Drug-coated balloon	44 (7.7)
Intraprocedural complications	35 (6.1)
Severe dissections (D - E - F)	10 (1.8)
Abrupt vessel closure	9 (1.6)
Perforation	8 (1.4)
Hemodynamic instability (intervention)	8 (1.4)
Complication IVL-related	5 (0.9)

Values are median (IQR) or n (%).

CTO = chronic total occlusion; IABP = intra-aortic balloon pump; IVL = intravascular lithotripsy; VA-ECMO = venoarterial extracorporeal membrane oxygenation.

**Table 3:** Technical and Clinical Outcomes (N = 571)

Technical success	511 (89.5)
TLF	41 (7.2)
Cardiac death	17 (4.6)
TV-MI	17 (4.6)
ID-TVR	36 (6.3)

Values are n (%).

ID-TVR = ischemic-driven target vessel revascularization; TLF = target lesion failure; TV-MI = target-vessel myocardial infarction; TVR = target vessel revascularization.

**Table 4:** Determinants of Technical Failure

Determinants	Univariate Analysis		Multivariate Analysis	
	OR (95% CI)	*P* Value	OR (95% CI)	*P* Value
Age	1.02 (0.98-1.05)	0.28	-	-
Sex	0.99 (0.58-1.77)	0.98	-	-
Diabetes	1.26 (0.75-2.12)	0.39	-	-
Syntax score	1.01 (0.99-1.03)	0.62	-	-
Fluoroscopic calcification	1.33 (0.90-1.95)	0.15	-	-
eGFR	0.99 (0.98-1.01)	0.37	-	-
Clinical Pres	1.19 (0.99-1.43)	0.06	-	-
Number of vessels disease	1.41 (1.03-1.91)	**0.03**	1.39 (1.02-1.90)	**0.035**
LM	0.87 (0.38-2.00)	0.76	-	-
Bifurcation	0.66 (0.34-1.31)	0.24	-	-
CTO	1.03 (0.42-2.52)	0.95	-	-
Ostial	1.48 (0.85-2.58)	0.17	-	-
Long lesions (>20 mm)	0.58 (0.34-0.97)	**0.04**	-	-
Tortuosity	2.27 (0.61-8.45)	0.22	-	-
In-stent	1.86 (1.10-3.15)	**0.02**	1.94 (1.14-3.30)	**0.015**
No ICI	1.98 (1.15-3.41)	**0.01**	1.95 (1.13-3.40)	**0.017**

**Bold** values indicate statistical significance.

eGFR = estimated glomerular filtration rate; ICI = intracoronary imaging; LM = left main artery; other abbreviation as in Table 2.

**Table 5:** Determinants of Target Lesion Failure

Determinants	Univariate Analysis		Multivariate Analysis	
	HR (95% CI)	*P* Value	HR (95% CI)	*P* Value
Age (per year increase)	0.97 (0.93-0.99)	**0.04**	0.95 (0.92-0.98)	**<0.01**
Sex (female)	0.69 (0.36-1.33)	0.25	-	-
Diabetes	2.21 (1.20-4.09)	**0.01**	2.35 (1.23-4.47)	**<0.01**
LVEF	0.95 (0.91-0.99)	**0.03**	-	-
Syntax score	1.01 (0.98-1.03)	0.47	-	-
Fluoroscopic calcification	1.39 (0.88-2.16)	0.17	-	-
eGFR	0.98 (0.97-0.99)	**0.03**	0.98 (0.97-0.99)	**0.02**
Number of vessels disease	1.32 (0.91-1.92)	0.14		
Technical failure	2.95 (1.53-5.81)	**<0.01**	2.42 (1.26-4.61)	**<0.01**
Left circumflex	2.59 (1.32-5.08)	**<0.01**	2.97 (1.49-5.92)	**<0.01**
Bifurcation	1.05 (0.50-2.20)	0.90	-	-
CTO	1.84 (0.92-5.00)	0.20	-	-
Ostial	1.64 (0.86-3.12)	0.13	-	-
Long lesions (>20 mm)	0.83 (0.44-1.59)	0.58	-	-
Tortuosity	2.31 (0.56-9.60)	0.24	-	-
In-stent	2.02 (1.13-3.84)	**0.02**	2.01 (1.02-3.52)	**0.04**

**Bold** values indicate statistical significance.

Abbreviations as in Tables 1, 2 and 4.

## Discussion

**Figure 2:**
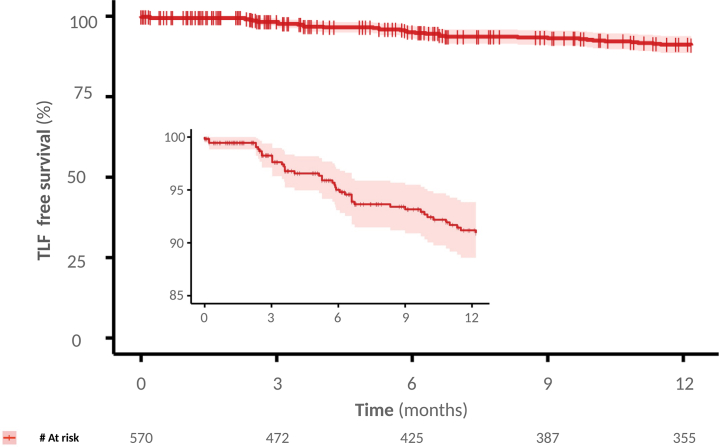
Kaplan-Meier Estimates of 12-Month Target Lesion Failure-Free Survival TLF = target lesion failure.

The primary objective of the present study was to identify determinants of technical failure and TLF in calcified coronary lesions treated with IVL. The incidence of technical failure (10.5%) and 12-month TLF (7.2%) was low. The main findings are as follows: 1) in-stent restenosis (ISR), absence of intravascular imaging, and multivessel disease were independent determinants of technical failure; and 2) younger age, DM, reduced estimated glomerular filtration rate, ISR, left circumflex target vessels, and technical failure were independently associated with TLF (Figure 2, Central Illustration).

**Central Illustration:**
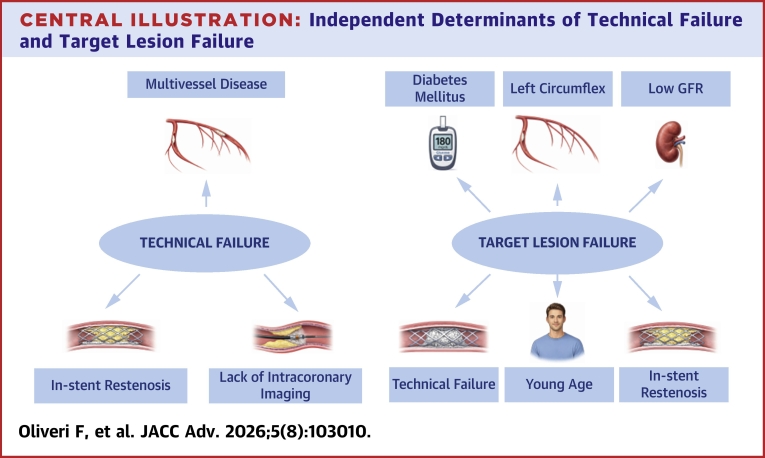
Independent Determinants of Technical Failure and Target Lesion Failure GFR = glomerular filtration rate.

### Anatomical and lesion-specific determinants: ISR and left circumflex artery

IVL pulses administrations in ISR were independently associated with both technical failure and long-term TLF. The metallic struts of the pre-existing scaffold can create acoustic shadowing, reflecting sonic pressure waves, and suboptimal dispersing energy to the underlying calcified plaque, potentially representing a mechanistic explanation. Moreover, ISR is frequently driven by fibrous or fibrocalcific neointimal hyperplasia.^13^^,^^14^ Because IVL selectively fractures calcium with virtually no effect on yielding fibroelastic tissue, its use is not enough to optimize lesion preparation.^13^^,^^14^ This highlights the need for a synergistic approach, potentially incorporating complementary devices (scoring or cutting balloons etc) alongside IVL to correctly modify the fibrous plaque components when ISR is the target.

Interestingly, left circumflex artery (LCx) artery lesions were independently associated with a higher risk of TLF. Compared with other epicardial vessels, the LCx frequently exhibits an angulated take-off and greater proximal tortuosity, which inherently reduces guide catheter support and impairs the deliverability of bulky calcium-modification tools, high-pressure balloons, and intravascular imaging catheters.^15^ Consequently, optimal lesion preparation is more difficult to achieve, drastically increasing the likelihood of recoil and residual stent underexpansion and/or malapposition. Nevertheless, calcified LCx sometimes involves ostial and bifurcation segments, including distal left main.^16^ When calcified nodules involve the continuity between the distal left main and the ostial LCx, procedural challenges and the risk of potential complications are further amplified.^16^

### Clinical determinants: DM, chronic kidney disease, and younger age

DM is a well-established independent determinant of TLF after PCI, manifesting as higher rates of ISR and major adverse cardiovascular events compared to nondiabetic patients.^17^^,^^18^ Uncontrolled DM promotes diffuse and complex coronary disease, increased intimal hyperplasia, vascular inflammation, endothelial dysfunction, and a prothrombotic state, all of which contribute to adverse outcomes.^17^^,^^18^ Although IVL effectively improves coronary artery compliance, favoring good SE, it does not mitigate the underlying systemic proinflammatory and prothrombotic milieu driving restenosis in these complex populations, explaining why DM remains independently associated with TLF.^6^^,^^7^

Despite the well-known clinical association between chronic kidney disease and DM, a reduced estimated glomerular filtration rate maintained an independent inverse association with TLF (demonstrated by low variance inflation factor during analysis). Large registry-based studies demonstrate that patients with chronic kidney disease have a significantly strong correlation with CAC and present higher cumulative incidence of TLF compared to those with preserved renal function.^19^ This risk is driven by increased rates of cardiac death and target lesion revascularization and is directly proportional to the severity of the renal impairment.^19^^,^^20^

Interestingly, younger patients displayed higher odds of TLF in our cohort. Although this finding may appear counterintuitive, it should be considered hypothesis-generating and interpreted with caution, as it likely reflects a complex interplay of biological and clinical factors. We hypothesize that 3 distinct mechanisms may contribute to this observation. First, in a cohort restricted to severely calcified lesions requiring IVL, premature presentation identifies a specific phenotype of biologically aggressive atherosclerosis, inherently characterized by a higher propensity for recurrent ischemic events. Second, elderly patients are disproportionately affected by competing noncardiovascular mortality, which may artificially attenuate their observed incidence of TLF over time (TLF does not include noncardiovascular death). Third, older patients are frequently managed conservatively upon symptom recurrence, whereas younger individuals present a lower clinical threshold for invasive reassessment and clinically driven TLR.

### Procedural determinants: intracoronary imaging and technical success

Multiple recent randomized trials and meta-analyses demonstrate that ICI-guided PCI (using IVUS or OCT) is associated with significantly reduced rates of TLF, including lower risks of cardiac death, MI, stent thrombosis, and TLR, compared to angiography-guided PCI.^21^^,^^22^ Thus, unsurprisingly, in our registry, the absence of ICI guidance was identified as a strong independent determinant of technical failure. Without imaging, operators cannot ensure the critical 1:1 IVL balloon-to-artery ratio required for optimal sonic energy transfer, nor can they definitively ensure adequate SE and apposition. Consequently, technical failure also emerged as a robust, independent determinant of TLF. Inadequate lesion preparation or incomplete SE inherently leads to stent thrombosis and ISR.^3^ Crucially, unlike systemic comorbidities, residual stenosis represents a fully modifiable procedural determinant, emphasizing the absolute need for meticulous optimization.

Currently, a dedicated clinical risk score for predicting late adverse events in patients undergoing IVL-assisted PCI is lacking in contemporary practice. The consistent associations observed in this analysis suggest that these variables may represent clinically relevant markers of adverse outcomes in patients undergoing PCI for heavily calcified coronary lesions. These findings may provide a foundation for future prospective studies aimed at developing simplified tools to support risk assessment, patient selection, and tailoring of adjunctive therapies.

### Study Limitations

The present study has limitations. First, as an observational study, we cannot entirely exclude the presence of unmeasured confounding factors inherent to its nonrandomized design. In particular, operator-dependent choices regarding adjunctive devices or strategies may have introduced a treatment-selection bias that multivariable adjustment can only partially mitigate Second, although our models showed good discriminatory performance and were internally validated through bootstrapping, the absence of external validation in an independent cohort remains a key limitation. Third, although the rate of ICI in our study is relatively high compared with routine real-world practice (54.5%), further increasing its use remains desirable.

## Conclusions

Our findings suggest that residual risk following IVL-assisted PCI is driven by a combination of modifiable and nonmodifiable factors rather than intrinsic limitations of the technology itself. Multivessel disease, in-stent lesions, and the absence of intravascular imaging are independently associated with technical failure. At 12 months, technical failure, DM, in-stent lesions, and left circumflex target vessels were determinants of TLF, reinforcing the importance of meticulous lesion preparation and imaging-guided PCI to optimize outcomes in heavily calcified coronary lesions.

Perspectives **COMPETENCY IN PATIENT CARE AND PROCEDURAL SKILLS:** While intravascular lithotripsy (IVL) is highly effective for calcified coronary lesions, residual risk is driven by a distinct interplay of modifiable and nonmodifiable factors rather than intrinsic limitations of the technology. To optimize patient outcomes, interventional cardiologists must prioritize meticulous lesion preparation and routinely utilize intracoronary imaging, as its absence is an independent determinant of technical failure. Furthermore, heightened procedural vigilance is required when treating complex anatomical subsets, such as in-stent restenosis or left circumflex artery lesions, which are independently associated with an increased risk of target lesion failure.

**TRANSLATIONAL OUTLOOK:** The identification of specific anatomical, procedural, and clinical predictors of technical and target lesion failure provides a critical foundation for personalized risk stratification in IVL-assisted percutaneous coronary intervention. Future prospective studies should aim to develop simplified, dedicated clinical risk scores to refine patient selection and support clinical decision-making. Additionally, further research is needed to evaluate whether tailored adjunctive therapies, such as combining IVL with complementary plaque-modifying devices, can effectively mitigate the higher risk of restenosis observed in challenging scenarios like in-stent lesions.

## Funding support and author disclosures

This work was funded through a research grant from 10.13039/100031956Shockwave Medical. Funders were not involved in any aspect of the study, including its design, collection, analysis and interpretation of the data, or the writing of the manuscript. The Department of Cardiology of the Leiden University Medical Center received unrestricted research grants from 10.13039/100011949Abbott Vascular, 10.13039/100004326Bayer, 10.13039/501100005035Biotronik, 10.13039/100008497Boston Scientific, 10.13039/100006520Edwards Lifesciences, GE Healthcare and 10.13039/100004374Medtronic. B.E.P.M. Dr Paradies received unrestricted research grants from 10.13039/100011949Abbott Vascular, SMT, and Terumo via the Institution; and consultancy fee from 10.13039/100011949Abbott Vascular, Novo Nordisk, SMT, Elixir Medical, and 10.13039/100008497Boston Scientific. Dr Claessen received consultancy fees from Abiomed, 10.13039/100011949Abbott Vascular, Amgen, BBraun, 10.13039/100008497Boston Scientific, Philips, and Sanofi; and received research funding from Philips, Novo Nordisk, BBraun, and Infraredx. Dr van der Kley received consultancy fees from 10.13039/100006520Edwards Lifesciences and 10.13039/100011949Abbott Vascular. Dr Jukema/his department has received research grants from and/or was speaker (with or without lecture fees) on a.o.(CME accredited) meetings sponsored/supported by Abbott, Amarin, Amgen, Athera, 10.13039/501100005035Biotronik, 10.13039/100008497Boston Scientific, Dalcor, Daiichi Sankyo, 10.13039/100006520Edwards Lifesciences, GE Healthcare Johnson and Johnson, Lilly, 10.13039/100004374Medtronic, Merck-Schering-Plough, Novartis, Novo Nordisk, Pfizer, Roche, Sanofi Aventis, Shockwave Medical, the Netherlands Heart Foundation, CardioVascular Research the Netherlands (CVON), the Netherlands Heart Institute, and the European Community Framework KP7 Programme. Dr Montero received a research grant from Shockwave Medical and speaker fees from Abiomed, 10.13039/100008497Boston Scientific and Penumbra Inc. All other authors have reported that they have no relationships relevant to the contents of this paper to disclose.
