# Supplementary material for: Technical and Target Lesion Failure in Calcified Coronary Lesions: Residual Risk Beyond Successful Lithotripsy
Source: JACC Adv. 2026 Jul 15;5(8):103010. doi: 10.1016/j.jacadv.2026.103010 (PMC13383997; doi:10.1016/j.jacadv.2026.103010)
Supplement: Supplemental Table 1 [file mmc1.docx]

| **Table S1 Intracoronary imaging characteristics** | |
| --- | --- |
|  | **Total population** (571) |
| **Intracoronary Imaging used** | 311/571 (54.5) |
| **Intracoronary imaging devices**  IVUS  OCT | 283/311 (92.9)  29/311 (7.1%) |
| **Reference vessel diameter (**mm) | 4.0 [3.6-4.4] |
| **Pre-IVL Minimum lumen diameter** (mm) | 1.9 [1.7-2.2] |
| **Pre-IVL Minimum lumen area** (mm^2^) | 3.8 [2.7-4.7] |
| **Pre-IVL Diameter stenosis** (%) | 51 [44-60] |
| **Pre-IVL Area stenosis** (%) | 70 [60-81] |
| **Max persistent Ca^2+^ angle** (^o^) | 360 [300-360] |
| **Post Minimum stent/lumen area** (mm^2^) | 9.5 [7.6-11.2] |
| **Post Stent expansion at MSA** (%) | 78 [68-85] |
| **Post Asymmetricity index at MSA** | 0.14 [0.08-0.22] |
| **Persistent Ca^2+^ fracture** | 183/311 (58.8) |

*Values are median (IQR) or n (%). MSA = minimum stent area.*
